# The True Challenges of the Covid-19 Epidemics: The Need for Essential Levels of Care for All

**DOI:** 10.2174/1874306402014010008

**Published:** 2020-03-16

**Authors:** Mauro Giovanni Carta, Ferdinando Romano, Germano Orrù

**Affiliations:** 1 University of Cagliari, Cagliari, Italy; 2 University of Rome La Sapienza, Rome, Italy

**Keywords:** Covid19, Public Health, Epidemics, Global Health, Essential level of care

In the last decades, biomedical research and funding supporting it have given a relevant impulse to developing so-called “Personalized Medicine” (PM) and “precision medicine” (the latter definition with emphasis on the usefulness of dividing patients into target groups). This perspective has increased knowledge on how we can predict disease susceptibility and prognosis in a person or how we can define a tailor-made treatment on specific individual immune-genomic characteristics and disease and thus improve the health of such a person [1].

All this represents an exhilarating challenge that has led to the discovery of treatments of unimaginable efficacy up to a few years ago. However, this recent advance in research seems to have had a price. In most countries, care of excellence is only for those who can afford it, but even in countries with strong national health systems, the commitment to indivi-dualized care may have diverted resources and attention from creating systems that guarantee protection for all. A health system that ensures well-being for all should not be in contradiction but rather complementary to medicine that tends to individualize treatments of excellence. Nevertheless, it is undeniable that the attention of researchers in recent years has not focused on public health and the sustainability of everyone's well-being.

In fact, several leading causes of mortality remain unaffected by PM [2]. It is well known that Infections of the respiratory tract are a major cause of morbidity and mortality worldwide [3]. If also in this field precision medicine has significantly contributed to improving disease prognosis [4], three successive coronavirus epidemics (SARS, MERS and COVID-19) have shown how deficiencies in providing an immediate and integrated medical responses, a certain inability to manage information and coordinate responses at local, natio- nal and international levels can favor the outbreak of epidemics that could be managed with less impact [5, 6].

In conclusion, it appears that something essential is missing: health services capable of responses for all, ability to correctly communicate health information, and skills in integrating care at various levels.

On the other hand, another awareness is emerging. That is, in the face of an epidemic, especially a respiratory epidemic with a strong infectious capacity (although in this case with low lethality), there can be no privileged areas immune from infection on the basis of one's own well-being and wealth. The Covid-19 has hit middle-high-class of people on a cruise and Wuhan and wealthy Lombardy cities in Italy, as well as suburban areas of the same Asian and European metropolises. Today there are reasons to fear that the impact and invasiveness of the epidemic could be amplified by the contagion of the population groups without assistance or with low-level assistance in rich nations and of low-income nations without a minimum health system.

This means that in terms of public health, defending the health of deprived areas and creating a network with a minimum level of assistance for all means prevention for everyone.

This is not to deny the importance of individualized medicine or of the great innovations that healthcare has introduced in these fields. But we must learn from this lesson that if we fail to guarantee a minimum support for everyone in terms of health services, prevention and treatment, we risk endangering everyone's health, not only that of people without privileges.
